# Minimizing shrinkage of acute brain slices using metal spacers during histological embedding

**DOI:** 10.1007/s00429-020-02141-3

**Published:** 2020-09-12

**Authors:** Felix Bolduan, Sabine Grosser, Imre Vida

**Affiliations:** Institute of Integrative Neuroanatomy, Charité - Universitätsmedizin Berlin, Berlin, Germany

**Keywords:** Acute brain slice, Single cell morphology, Biocytin labeling, Histology, Tissue shrinkage, Confocal imaging

## Abstract

The morphological structure of neurons provides the basis for their functions and is a major focus of contemporary neuroscience studies. Intracellular staining of single cells in acute slices is a well-established approach, offering high-resolution information on neuronal morphology, complementing their physiology. Despite major technical advances, however, a common histological artifact often precludes precise morphological analysis: shrinkage of the sampled tissue after embedding for microscopy. Here, we describe a new approach using a metal spacer, sandwiched between two coverslips to reduce shrinkage of whole-mount slice preparations during embedding with aqueous mounting medium under a coverslip. This approach additionally allows imaging the slices from both sides to obtain better quality images of deeper structures. We demonstrate that the use of this spacer system can efficiently and stably reduce the shrinkage of slices, whereas conventional embedding methods without spacer or with agar spacer cause severe, progressive shrinkage after embedding. We further show that the shrinkage of slices is not uniform and artifacts in morphology and anatomical parameters produced cannot be compensated using linear correction algorithms. Our study, thus, emphasizes the importance of preventing the deformation of slice preparations and offers an effective means for reducing shrinkage and associated artifacts during embedding.

**Electronic supplementary material:**

The online version of this article (10.1007/s00429-020-02141-3) contains supplementary material, which is available to authorized users.

## Introduction

The morphology of neurons provides the structural framework for their functions, including the integration of synaptic inputs and the generation of action potentials (Kasper et al. 1994; Norenberg et al. 2010; DeFelipe et al. 2013; de Sousa et al. 2015; Gulledge and Bravo, 2016; Mihaljevic et al. 2018). Indeed, from Cajal’s morphological studies of Golgi stained neurons (Ramón y Cajal 1909, 1911) up to today’s high-resolution confocal images of genetically identified and biocytin stained neurons (Bartos et al. 2002; Thomson and Armstrong 2011; Booker et al. 2014), anatomical investigations offered important insights for the understanding of the physiological and circuit functions of neurons and also provided essential data for computational analysis (Traub et al. 1994; Major et al. 1994; Norenberg et al. 2010; Gidon et al. 2020). While identification, labeling and imaging of neurons have shown substantial advances in recent decades, a major technical problem of morphological analysis remains: the shrinkage of tissue during histological processing and embedding.

Processing of tissue sections or acute slice material by applying conventional methods leads to a strong reduction in their overall dimensions, in particular, in their thickness along the ‘*z*-axis’ (the axis perpendicular to the cut surfaces of the slice or section). This deformation of the tissue has a substantial impact on the morphology of the neurons observed under the microscope and, consequently, also on their reconstructions and derived anatomical parameters. In fact, *z*-shrinkage has been previously recognized and found to be highly dependent on the histological processing and embedding: histological procedures which include drying or dehydration of the tissue cause severe shrinkage (Pyapali et al. 1998; Hellwig 2000; Marx et al. 2012). Avoiding such steps in the processing and the use of aqueous mounting media can help to eliminate shrinkage during processing; nevertheless the slices show shrinkage after they are embedded under a coverslip (Egger et al. 2008; Swietek et al. 2016).

To prevent this shrinkage, agar spacers between glass slide and coverslip were used in some studies (Booker et al. 2014; Degro et al. 2015). However, the long-term stability of this soft spacer has not been examined. In fact, the process of shrinkage in general has remained poorly characterized. Its precise degree and time course are not known as previous investigations examined only a single and often not well-defined time point (Pyapali et al. 1998; Egger et al. 2008). Furthermore, the impact of shrinkage on neuronal morphology and anatomical parameters are unknown: assuming a uniform, linear shrinkage, correction factors have been employed to three-dimensional (3D) reconstructions in some studies in order to compensate for the distortion effects (Pyapali et al. 1998; Hellwig 2000; Degro et al. 2015). However, there is no evidence that such compensation is, indeed, an adequate approach for shrinkage correction.

Here, we describe a new approach using a metal spacer to minimize tissue shrinkage in fixed brain slices. By comparing embedding with metal spacers to other commonly used methods we demonstrate that metal spacers markedly reduce tissue shrinkage over time. Our analysis further reveals that z-shrinkage during conventional embedding produces non-uniform distortions in the slices and neuronal morphology, which cannot be adequately compensated by common linear methods. Thus, prevention of tissue shrinkage is essential in minimizing measurement errors in anatomical parameters in mounted slices.

## Methods

### Slice preparation

Experiments and animal maintenance were performed in accordance with local (LaGeSo, Berlin, T 0215/11), national (German Animal Welfare Act) and international guidelines (EU Directive 2010/63/EU). For obtaining whole-cell recordings and examining the shrinkage of acute brain slices, rat hippocampal slices were made and processed as previously described (Booker et al. 2014). In brief, brains were quickly dissected from decapitated rats (male and female Wistar-VGAT-Venus-A rats, P21–P25) after deep isofluran anesthesia (3%) and were put into carbogenated (95% O_2_/5% CO_2_), semi-frozen, sucrose-based artificial cerebrospinal fluid (sACSF, in mM: 87 NaCl, 2.5 KCl, 25 NaHCO_3_, 1.25 NaH_2_PO_4_, 25 Glucose, 75 Sucrose, 1 Na_2_-Pyruvate, 1 Na_2_-Ascorbate, 7 MgCl_2_, 0.5 CaCl_2_). Horizontal slices (300 µm) were subsequently cut from the hippocampal formation using a vibratome (VT1200s, Leica, Germany). The slices were transferred to carbogenated sACSF (34 °C) and left there for 30 min to recover. Afterwards, the slices were stored at room temperature in sACSF until further procedures. For analysis of shrinkage, slices were directly fixed with 4% paraformaldehyde (PFA) in 0.1 M phosphate buffer (PB) overnight at 4 °C.

### Whole-cell recording, intracellular filling and visualization of neurons

To examine the impact of shrinkage on neuronal morphology, whole-cell patch-clamp recordings were performed in combination with intracellular filling in a submerged recording chamber, perfused with carbogenated artificial cerebrospinal fluid (ACSF, in mM: 125 NaCl, 2.5 KCl, 25 NaHCO_3_, 1.25 NaH_2_PO_4_, 25 Glucose, 1 Na_2_-Pyruvate, 1 Na_2_-Ascorbate, 1 MgCl_2_, 2 CaCl_2_) at a temperature of about 32 °C. Subicular pyramidal cells were visualized using an upright microscope (BX-51WI, Olympus, Hamburg, Germany) equipped with a digital camera (Zyla 5.5 sCMOS, Andor—Oxford Instruments, Abingdon, UK). Recordings were performed using a Multiclamp 700B amplifier (Molecular Devices, San Jose, CA). Patch pipettes were pulled from borosilicate glass capillaries (2 mm outer/1 mm inner diameter, Hilgenberg, Germany) on a horizontal electrode puller (P-97, Sutter Instruments, Novato, CA) and filled with an intracellular solution (in mM: 130 k-gluconate, 10 KCl, 2 MgCl_2_, 10 EGTA, 10 HEPES, 2 Na_2_-ATP, 0.3 Na_2_-GTP, 1 Na_2_-creatinine, and 0.1% Biocytin; 290–310 mOsm). A whole-cell recording was performed for at least 16 min to ensure sufficient diffusion of the biocytin and filling the cell’s somato-dendritic domain (Marx et al. 2012).

At the end of the recording, the pipette was carefully removed from the cell to form an outside-out patch and the slice instantly transferred to a 4% PFA containing 0.1 M PB based solution. After overnight fixation at 4 °C, slices were repeatedly rinsed in PB for at least 1 h and incubated with AlexaFlour 647-conjugated streptavidin (1:1000, Invitrogen, Eugene, OR) diluted in 0.1 M PB containing 0.05% NaN3 and 0.5% Triton-X overnight at 4 °C. The slices were subsequently rinsed repeatedly in PB before embedding.

### Embedding procedures for acute slices

**Fig. 1:**
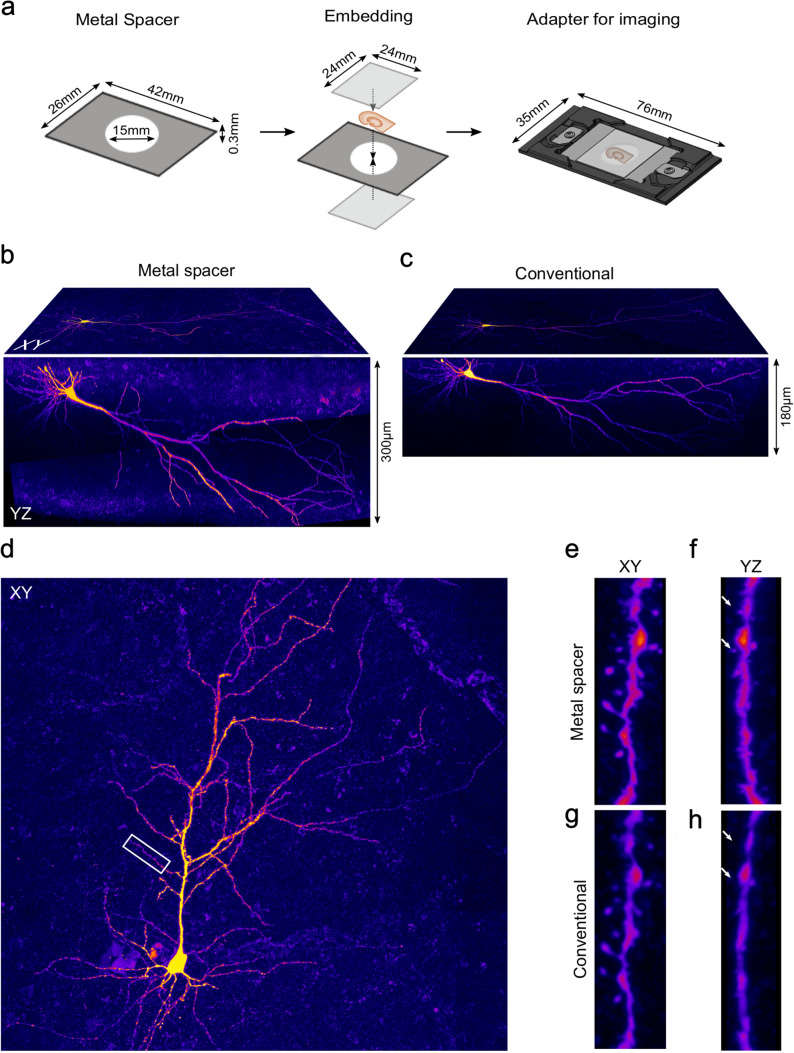
Embedding of acute slices with the metal spacer system for light microscopic analysis minimizes shrinkage and improves image quality. **a** Schematic representation of the embedding process using the metal spacer system. The 300 µm thick metal spacer (left) and a fixed brain slice are sandwiched between two glass coverslips with a solidifying aqueous mounting medium (middle). A custom-made adapter is required for imaging the embedded slices (right). **b, c** Orthogonal projections of a full confocal image stack of a subicular pyramidal neuron onto the *xy*- (top surface) and the *yz*-planes (front surface) embedded with a metal spacer (**b**) and following the conventional embedding approach without a spacer (**c**). Note the substantial shrinkage and the weak signal in the deeper parts of the slice after conventional embedding. **d** Projection of the confocal image stack of another subicular pyramidal neuron onto the *xy*-plane embedded with a metal spacer. **e**–**h** High-resolution images of a dendritic segment (rectangle in **d**) embedded first with the metal spacer (**e**, **f**) and after re-embedding following the conventional approach without a spacer (**g**, **h**) viewed in *xy*- (**e**, **g**) and in *yz*-projections (**f**, **h**). The image in panel **h** was compensated for shrinkage for better comparison with panel **f**. Note the reduced detail of the contours of the dendrite and the absence of some spines (arrows in **h** and **g**) in the *yz*-plane after conventional embedding

Slices were embedded in a solidifying aqueous mounting medium (Fluoromount-G, Southern Biotech, Birmingham, AL) using three different approaches:

1. For the conventional embedding method, the slices were mounted on a standard glass slide without any spacer and a coverslip placed on top. Care was taken to mount the slices with the recorded neurons (i.e. their somata) closer the upper surface when placed on the glass slides. Finally, coverslips were sealed with nail polish.
2. To prevent shrinkage of the mounted slice, the conventional embedding procedure was modified by adding a 300 µm thick agar spacer between slide and coverslip (Booker et al. 2014; Degro et al. 2015). To produce these spacers, agar (4%; Formedium Ltd, Hunstanton, UK) was diluted in 0.1 M PB and heated up to 90 °C. After cooling down, a solid block was formed, which was cut to the size of the coverslips (24 × 24 mm) and sectioned at 300 µm on a vibratome. Finally, a round hole with a diameter of 8 mm was stamped into the middle of the spacers. The spacers were collected and stored in 0.1 M PB until usage to prevent them from drying out.
3. In the new embedding approach, the agar spacer was replaced by a 300 µm thick metal spacer (Fig. 1). The metal spacer was made of stainless steel and had rectangular form with measures also defined by the coverslips, but exceeding those by a few millimeters in all directions (42 × 26 mm) to enable a stable contact and sealing with nail polish. The spacer had a round hole with a diameter of 15 mm in their center. The spacers were sourced from a commercial provider for custom-made metal objects (wh Münzprüfer, Berlin, Germany). For the embedding, one coverslip was glued onto the metal spacer using cyanoacrylate (UHU, Bühl, Germany), serving as a bottom plate during the mounting. After mounting the slice in the aqueous mounting medium, a second coverslip was carefully placed on the top of the assembly, making sure that no air was trapped, and sealed with nail polish.

### Confocal imaging, 3D reconstruction and morphological analysis of neurons

Imaging of neurons was performed using a laser scanning confocal microscope (FluoView FV1000, Olympus, Hamburg, Germany) with a 30 × silicone oil-immersion objective (N.A. 1.05; Olympus). To image slices embedded with metal spacers on the microscope stage, a custom-made adapter (Fig. 1a) was obtained from the commercial provider (wh Münzprüfer). Image stacks were acquired at a *xy*-resolution of 1024 × 1024 (2 µs pixel dwell time; pixel size: 0.414 µm) and 0.5 µm steps along the vertical *z*-axis. To improve imaging for deeper parts of the slices embedded with metal spacers, they were imaged first from the upper surface to the middle of the slice and subsequently from the bottom surface to the middle of the slice by flipping them in the adapter for the microscope stage. For selected neurons, high-resolution images of dendritic segments were performed using a 60 × silicone oil-immersion objective (N.A. 1.3) at 2048 × 2048 resolution (2 µs pixel dwell time; pixel size: 0.207 µm) and 0.05 µm steps between imaging planes along the *z*-axis.

Obtained image stacks were registered and stitched using the ‘3D stitching’ plug-in (Preibisch et al. 2009) of the Fiji/ImageJ software package (https://fiji.sc/; Schindelin et al. 2012). 3D reconstructions were made from stitched image stacks using the ‘Simple neurite tracer’ plug-in (Longair et al. 2011) in Fiji or the Neutube software package (Feng et al. 2015). Reconstructed morphologies were stored as SWC files (Cannon et al. 1998).

Morphological analysis of the reconstructed neurons was performed in the NEURON software environment (Hines and Carnevale, 1997) using custom written ‘hoc’ scripts (Degro et al. 2015). Neuronal morphologies were imported using the ‘import3d’ tool package. To reduce the raggedness of reconstructed neuronal process trajectories, which is inherent to reconstructions made by the ‘Simple neurite tracer’ plug-in, in particular along the *z*-axis, a Gaussian spatial filter was applied (3-point window, single run in the *xy*-plane and 15 iterations for values along the *z*-axis). Z-shrinkage compensation was accomplished by using a correction factor applied to the *z*-coordinates of the reconstructed neuronal structure. The correction factor was determined by calculating the quotient of the original nominal thickness (300 µm) and the measured thickness of the shrunken slices.

### Measurement of z-shrinkage of embedded slices

The thickness of the embedded slice was measured on the laser scanning confocal microscope (FluoView FV1000, Olympus) with an 30 × objective. Boundaries of the slice could easily be identified due to the non-specific background fluorescence of the tissue. The table’s vertical travel between top and bottom views of the slice was measured and taken as the thickness (Dorph-Petersen et al. 2001). First measurements of slice thickness were made 3 h after embedding, followed by measurements at distinct time points for up to 2 months. The slice thickness was always measured in the same region, at the crest of the granular cell layer of the dentate gyrus. Shrinkage was calculated as the difference of the measured and the nominal thickness (300 µm) expressed as percentage of the nominal value.

### Analysis of the impact of shrinkage on the intracellular filled neurons

To analyze the impact of shrinkage with the different embedding approaches, slices (*n* = 6) were first embedded with a metal spacer, and subsequently re-embedded following the conventional approach without a spacer. The slices were imaged 2–5 days after embedding with the metal spacer, to obtain a set of reference images and reconstructions of the neurons in this minimally shrunken state. The second imaging session was performed 11–14 days after re-embedding without spacer, to obtain images and reconstructions in a strongly shrunken state. Comparative dendritic length measurements were made with and without linear compensation for shrinkage (Degro et al. 2015), performed on the reconstructed neurons in the NEURON software package (see above).

To assess the effect of embedding on the slices in the *xy*-plane, the *z*-values of the 3D neuronal structures were set to zero and the total dendritic lengths of the flattened neurons were determined for the two conditions. To directly measure dimensional changes in *xy*-plane, photomicrographs of slices (*n* = 4) were made in the recording chamber at day 4 after embedding with the metal spacer and subsequently 14 days after re-embedding the slices without a spacer. Measurements of distances between selected landmarks in the plane of the slices were made in these sets of photomicrographs and the changes in distances normalized to their values obtained in the recording chamber. Additionally, lengths of dendritic segments projected in *xy*-plane were measured and compared for the two embedding conditions: with metal spacer and after re-embedding without spacer.

To assess if shrinkage was uniform along the *z*-axis in the slices, pairs of image stacks were analyzed. Projections of the two sets of image stacks were made in the *yz*-plane and the *z*-dimension of corresponding dendritic segments measured in the upper, the middle and the lower thirds of the slices. The difference of the two measurements, normalized to the value of the first measurement, was taken as the degree of differential shrinkage. As slices embedded with the metal spacer showed a small (≤ 4%), but consistent shrinkage along the *z*-axis at the time point of the imaging, the values calculated are underestimates of the full scale of shrinkage. In view of the large difference, however, we consider these estimates as representative for the process.

### Statistics

Statistical analysis was performed using Graphpad Prism version 7 (GraphPad Software, San Diego, CA). Time series of slice thickness for the different embedding methods were compared with a two-way ANOVA for repeated measures. Comparisons of slice thickness for the three conditions at given time points as well as the degree of shrinkage in distinct depths of the slices were made using one-way analyses of variance (ANOVA) with Bonferroni correction for multiple comparisons. Paired data were compared with Wilcoxon signed rank tests. Values are indicated as mean ± SD throughout. Statistical significance was assumed if *p* < 0.05.

## Results

### Description of the metal spacer system

To minimize shrinkage along the *z*-axis of acute slices after embedding, we developed a method using a metal spacer (Fig. 1). The metal spacer had a thickness equal to that of the slices (nominal 300 µm) typically used in electrophysiological recordings and had a rectangular shape (42 × 26 mm), exceeding slightly the size of the coverslips (24 × 24 mm) (Fig. 1a). To provide sufficient space for mounting slices, we opted for a large, central round opening (15 mm). We chose a spacer design that permits slices to be mounted between two coverslips of high optical quality, rather than between a single coverslip and glass slide, which is the current standard. This design requires a special adapter for imaging on a regular microscope stage, but enables imaging of the embedded slice from both sides (Fig. 1a). Thus, a better signal can be achieved in the depth and imaging of the full extent of the slice is possible when using objectives with short working distances by flipping the slices embedded with the metal spacer in the adapter.

Indeed, slices embedded using the metal spacer preserved their thickness well and showed a higher intensity of labeled structures (e.g. dendrites) and a better signal to noise ratio in their bottom halves when imaged from both sides (Fig. 1b) in comparison to the image stacks made 10–14 days after re-mounting the slices conventionally on a commonly used glass slide without a spacer imaged from the top (Fig. 1c). Images of dendritic segments in slices embedded with the metal spacer also captured more detail of their morphology when compared to image stacks made after re-embedding with the conventional approach (Fig. 1d–h). While differences between the pairs of image sets were not prominent in the *xy*-plane (Fig. 1e, g), they were very obvious in side views, e.g. in projections onto the *yz*-plane, revealing more detail of the contours of dendrites and more spines above and below the dendritic segments with the metal spacer (Fig. 1f) than after re-embedding without a spacer (Fig. 1h). In fact, counting the number of spines on dendritic segments and calculating their density in projection onto the *xy*-plane delivered matching results for the two embedding states (0.85 ± 0.17 µm^−1^, vs. 0.8 ± 0.12 µm^−1^, 8 dendritic segments, for metal spacer and conventional embedding, respectively, *p* = 0.312, Wilcoxon signed rank test, Online Resource 1a, b), whereas the estimated density was markedly lower in projections onto the *yz*-plane with the conventional embedding (0.41 ± 0.18 µm^−1^) than with the metal spacer (0.62 ± 0.14 µm^−1^, *p* = 0.016, Wilcoxon signed rank test, Online Resource 1c,d). Convergently, when applying a 3-d counting approach in the corresponding image stacks, the spine density was underestimated by 16% in slices embedded with the conventional method (1.25 ± 0.19 µm^−1^ vs. 1.05 ± 0.16 µm^−1^, respectively, *p* = 0.0078, Wilcoxon signed rank test, Online Resource 1e), indicating that shrinkage-induced reduction in physical resolution along the optical axis can result in underestimates of spine densities in slices with conventional embedding.

### The metal spacer can minimize the shrinkage of embedded slices

**Fig. 2:**
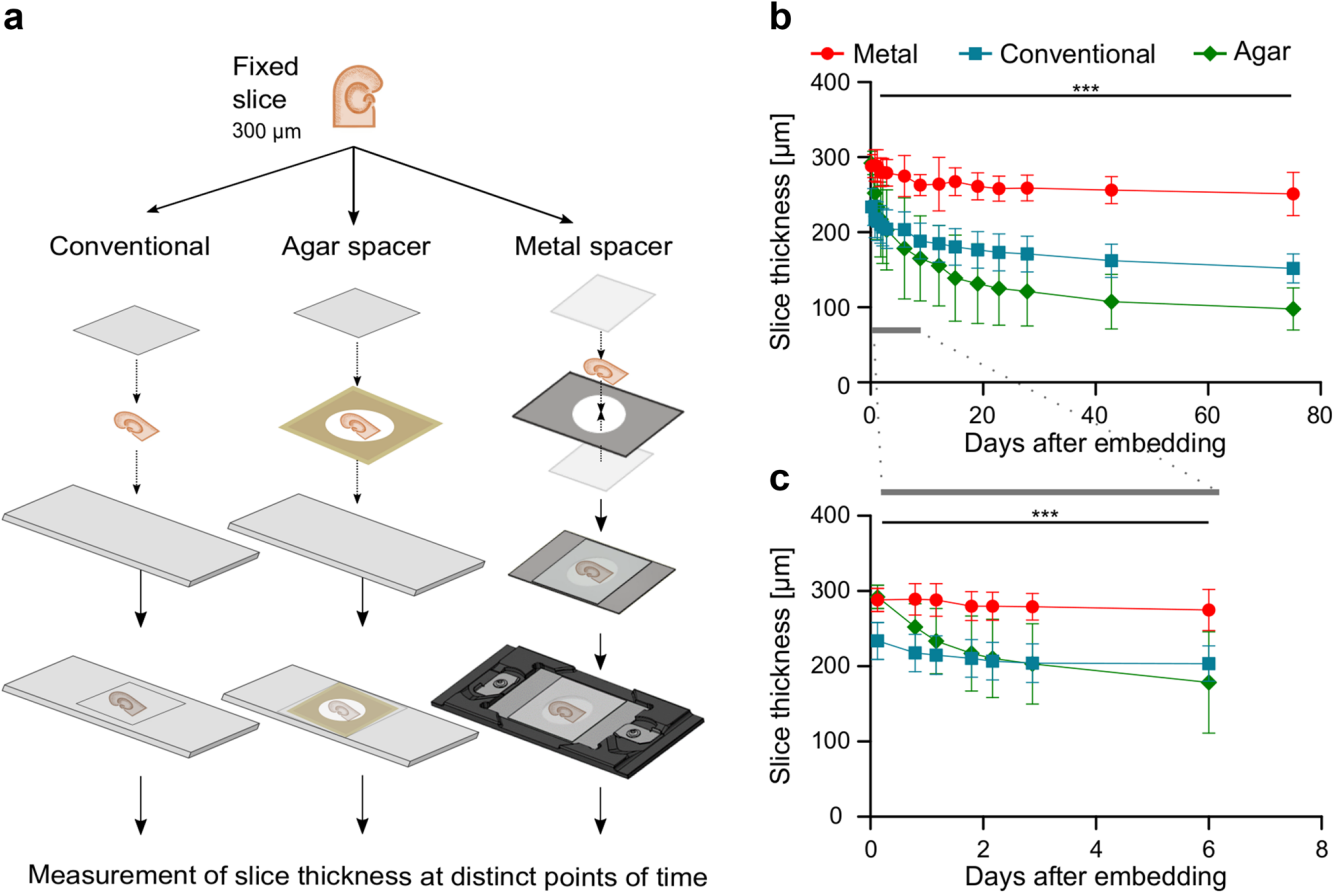
The rapid and strong shrinkage of fixed brain slices observed after conventional embedding is minimized by the metal spacer. **a** Schematic representation of the embedding of the acute slices using three alternative approaches: (1) conventional embedding method without any spacer on a normal glass slide with a coverslip (left arm), (2) a modified version using a 300 µm thick agar spacer between slide and coverslip (middle), and (3) using the metal spacer (right). **b** Summary graph of the average slice thickness plotted over time after embedding with the metal spacer (red circles, 8 slices), following the conventional approach without spacer (blue squares, *n* = 11), and with the agar spacer (green diamonds, *n* = 14). **c** The plot shows the same data at higher temporal resolution for the first 6 days (gray bar in **b**). Note, that the agar spacer initially prevents shrinkage of the slices, however, the thickness of these specimens rapidly decreases and declines below that of the slices without a spacer already beyond 3 days. Error bars indicate standard deviation; statistical significance: ***p < 0.001

To evaluate the long-term effectiveness of the metal spacer for preventing slice shrinkage, we next assessed the time course of changes in the thickness of acute slices (nominal 300 µm) embedded in a solidifying aqueous mounting medium using three different approaches (Fig. 2a): (1) without any spacer on a regular glass slide with a coverslip (conventional method), (2) a modified version using a 300 µm thick agar spacer between slide and coverslip, and (3) using the 300 µm thick metal spacer system. The first measurements of slice thickness were performed on a confocal microscope three hours after embedding (Fig. 2b). Slices embedded without spacer (*n* = 11) shrunk substantially by about 22.1 ± 8.1% in their thickness already at this early time point. In contrast, slices with metal (*n* = 8) or agar spacer (*n* = 14) showed only minimal shrinkage of 3.9 ± 4.8% and 2.6 ± 5.0% along the *z*-axis, respectively (*p* < 0.0001, ANOVA with Bonferroni correction for multiple comparisons). Thus, there is strong reduction in the thickness of the slices taking place in the first hours after embedding using the conventional approach, but both types of spacer are effective in minimizing this initial shrinking.

The shrinking process of the conventionally embedded slices continued in the following days and weeks, albeit at a progressively decelerating rate and approached asymptotic levels beyond 2 months (Fig. 2b). The thickness of the slices was found to be reduced by 49.4 ± 6.1% when measured 75 days after embedding (Fig. 2b). Slices embedded with the spacers also continued to shrink, but depending on the type, they showed highly divergent temporal dynamics and reached different asymptotic levels. Slices embedded with the metal spacer shrunk slowly and moderately, losing only 16.3 ± 8.9% of their thickness when measured after 75 days (Fig. 2b). In contrast, slices with agar spacer showed an accelerated and strong shrinkage, reaching a mean of 67.4 ± 9.0% by the same time point (Fig. 2b). In fact, although agar spacer could prevent the initial shrinkage, the reduction in the thickness of those slices progressed very rapidly in the first days (Fig. 2c) and was equal to that of conventional embedded slices already at day 3 (32.2 ± 17.1% vs. 31.9 ± 8.1%) and exceeded that by day 6 post-embedding (40.5 ± 21.6% vs. 32.1 ± 7.4%). In comparison, slices embedded with metal spacer showed a z-shrinkage of only 8.4 ± 8.5% at day 6 post-embedding (*p* = 0.0004, ANOVA with Bonferroni correction for multiple comparisons, Fig. 2c).

In summary, the use of metal spacers can efficiently minimize shrinkage, offering a major improvement over conventional embedding methods. In contrast, while agar spacer can prevent the initial shrinkage during the first hours after embedding, they are not useful to minimize shrinkage on longer time scales and, in fact, strongly promote shrinkage beyond 1 week.

### Shrinkage has an impact on reconstructed structure and anatomical parameters of labeled neurons

Prior morphological studies assumed a uniform slice shrinkage and used linear correction factors to compensate its impact on reconstructions (Pyapali et al. 1998; Hellwig, 2000; Marx et al. 2012). Therefore, we next addressed how the morphology of filled neurons changes due to shrinkage and whether it was possible to compensate their deformation by applying a linear correction factor.

**Fig. 3:**
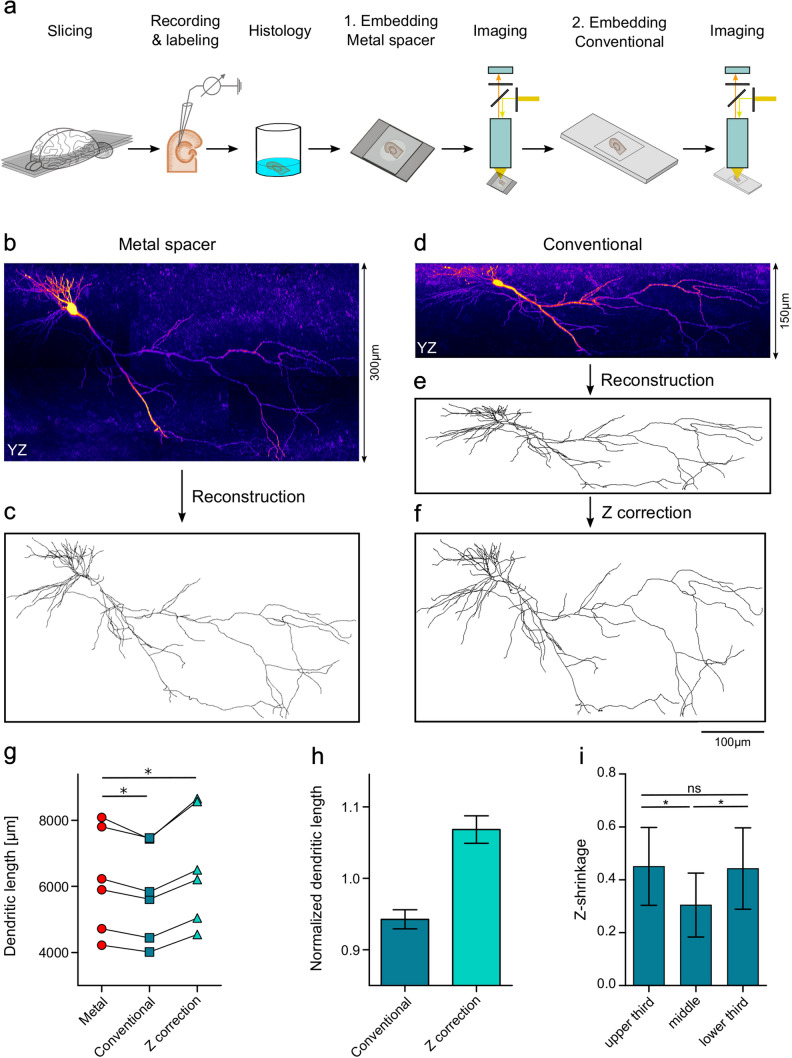
The impact of shrinkage on the morphology of intracellularly labeled neurons. **a** Schematic representation of the procedure analyzing the impact of shrinkage on neuronal morphology, which includes a first embedding with a metal spacer followed by confocal imaging and a re-embedding without a spacer (2. Embedding, Conventional) and repetition of the confocal imaging with a delay of 11–14 days. **b**–**f** Morphological data of a representative pyramidal cell under the different embedding conditions. *yz*-Projections of the confocal image stacks obtained from the slice embedded with the metal spacer (**b**) and after re-embedding without spacer (**d**). Full 3D reconstructions of the neuron viewed in the *yz*-projection made from the image stacks with metal spacer (**c**) and without spacer (**e**), and the latter reconstruction after correction for shrinkage along the *z*-axis (**f**). **g** Plot of the total dendritic lengths of 6 neurons measured in reconstructions made with metal spacer (red circles), in reconstructions made after re-embedding without spacer (blue squares) and after linear shrinkage correction was applied to the second set of reconstructions (cyan triangles). Lines connect data points representing measurements from the same neuron. **h** Summary bar chart of the dendritic lengths measured in the reconstructions made after conventional embedding (blue bar) and after linear shrinkage correction was applied (cyan bar), normalized to the corresponding lengths measured in reconstructions with metal spacer. **i** Summary bar chart of the degree of shrinkage of dendritic segments in the vertical dimension (z-shrinkage) in the upper, middle and lower thirds of slices. Measurements of the *z*-dimension of dendritic segments (15 dendrites from 3 slices for each depth) were made in image stacks obtained after conventional embedding and normalized to the corresponding values measured with the metal spacer. Error bars indicate standard deviation; statistical significance: *p < 0.05

To obtain neuronal morphologies, we performed whole-cell patch-clamp recordings in combination with intracellular biocytin labeling of pyramidal cells in 300 µm thick acute hippocampal slices (Fig. 3). After histological processing and visualization of the neurons, the slices were embedded first with a metal spacer (Fig. 3a) and imaged on a confocal microscope within 2–5 days (Fig. 3b). Neuronal morphologies were reconstructed using the simple neurite tracer plug-in (Longair et al. 2011) in the Fiji software package (Fig. 3c). Subsequently, the slices were removed from the metal spacer system and re-embedded following the conventional approach without a spacer (Fig. 3a); 11–14 days after re-embedding, the neurons were imaged (Fig. 3d) and reconstructed anew (Fig. 3e). This way we obtained full image sets and reconstructions of the somato-dendritic domains of six pyramidal cells in a minimally shrunken state (embedded with metal spacer) and a strongly shrunken state (conventional embedding). Finally, to compensate for the difference in the shrinkage between the two embedding states, a linear correction factor was applied to the *z*-coordinates of the second reconstruction (Fig. 3f). The correction factor was calculated as the quotient of the measured thickness for each slice mounted with metal spacer and the corresponding value obtained after re-embedding without spacer.

When visually compared, there were no major differences when the pairs of image stacks or 3D-reconstructions were projected onto the *xy*-plane (i.e. viewed from the top surface of the slices). In contrast, the impact of shrinkage on dendritic morphology was very obvious when the stacks and reconstructions were projected onto the *yz*-plane (i.e. viewed from the side, Fig. 3b, c versus Fig. 3d, e). Consistent with the differential shrinkage described above, the thickness of the slices after re-embedding without spacer was reduced by 42.0 ± 4.6% relative to the values measured during the first embedding using the metal spacer.

To analyze the impact of z-shrinkage on anatomical parameters, the total dendritic length of the neurons was calculated and compared between the two reconstructions for each neuron. This comparison demonstrated that the total dendritic lengths were consistently lower by a factor of 5.7 ± 1.2% on average in the reconstructions obtained after the re-embedding (*p* = 0.03, Wilcoxon signed rank test, Fig. 3g, h), confirming that shrinkage causes an alteration in morphological parameters. However, this difference was not corrected well when we attempted to compensate for shrinkage in the second set of reconstructions by applying the linear correction factor to the *z*-coordinates. With this correction, the total dendritic lengths were consistently higher by 6.8 ± 1.8%, than those obtained for the first reconstructions with the metal spacer (*p* = 0.03; Wilcoxon signed rank test, Fig. 3g, h).

To test if the reconstruction algorithm was not introducing errors in this evaluation, we generated a second set of reconstructions from the same image stacks using Neutube as reconstruction software (Feng et al. 2015). The total dendritic lengths measured in these reconstructions (Online Resource 2) were slightly smaller than those in the reconstructions made in Fiji, plausibly due to the lower level of spatial noise produced along the dendritic axes by the algorithms used in Neutube (see “Methods”). Nevertheless, when the second reconstructions of the neurons, obtained from the conventional embedding, were compared with the first ones, obtained from embedding with metal spacer, the total dendritic lengths were found again to be consistently smaller by a factor of 5.5 ± 1.3% on average (*p* = 0.03, Wilcoxon signed rank test, Online Resource 2a, b). Furthermore, the total dendritic lengths from the corrected reconstructions exceeded the corresponding values with metal spacer by 7.8 ± 2.0% (*p* = 0.03, Wilcoxon signed rank test, Online Resource 2a, b). These findings convergingly suggest that effects of shrinkage on the neuronal morphology cannot be properly compensated, plausibly, because the shrinkage is not uniform and/or the slices may suffer additional deformation during conventional embedding without a spacer.

### Shrinkage is not uniform across the depth of the slices

To investigate these possibilities, we examined first if there was any evidence for a non-uniform shrinkage. We made measurements of dendritic segments, which were primarily oriented along the *z*-axis, in the two pairs of confocal image stacks obtained with the metal spacer and after re-embedding. For each slice, three sets of samples were collected corresponding to the upper, the middle and the lower third along the *z*-axis of the slices in order to estimate and compare the degree of shrinkage at different depths. Evaluation of these samples showed that the shrinkage was affecting the *z*-dimension of dendritic segments on average by 45.1 ± 14.2% in the top, 30.4 ± 11.7% in the middle, and 44.3 ± 14.8% in the bottom third of the slices (*p* = 0.01, ANOVA with Bonferroni correction for multiple comparisons, 15 dendritic segments in each sample from 3 slices, Fig. 3i). Thus, the shrinkage was found to be stronger closer to the upper and lower surfaces of the slices than in their middle. To further confirm this notion, we made additional measurements in the top and bottom 20% of the slices and found that shrinkage in these marginal zones of the slices reached 61.3 ± 9.1% (ten dendritic segments in the top or bottom 20% in two slices). These results clearly indicate a non-uniform, non-linear shrinkage of the slices along the *z*-axis, which renders linear compensation impossible.

### Shrinkage is accompanied by a moderate dilation in the *xy*-plane of the slices

**Fig. 4:**
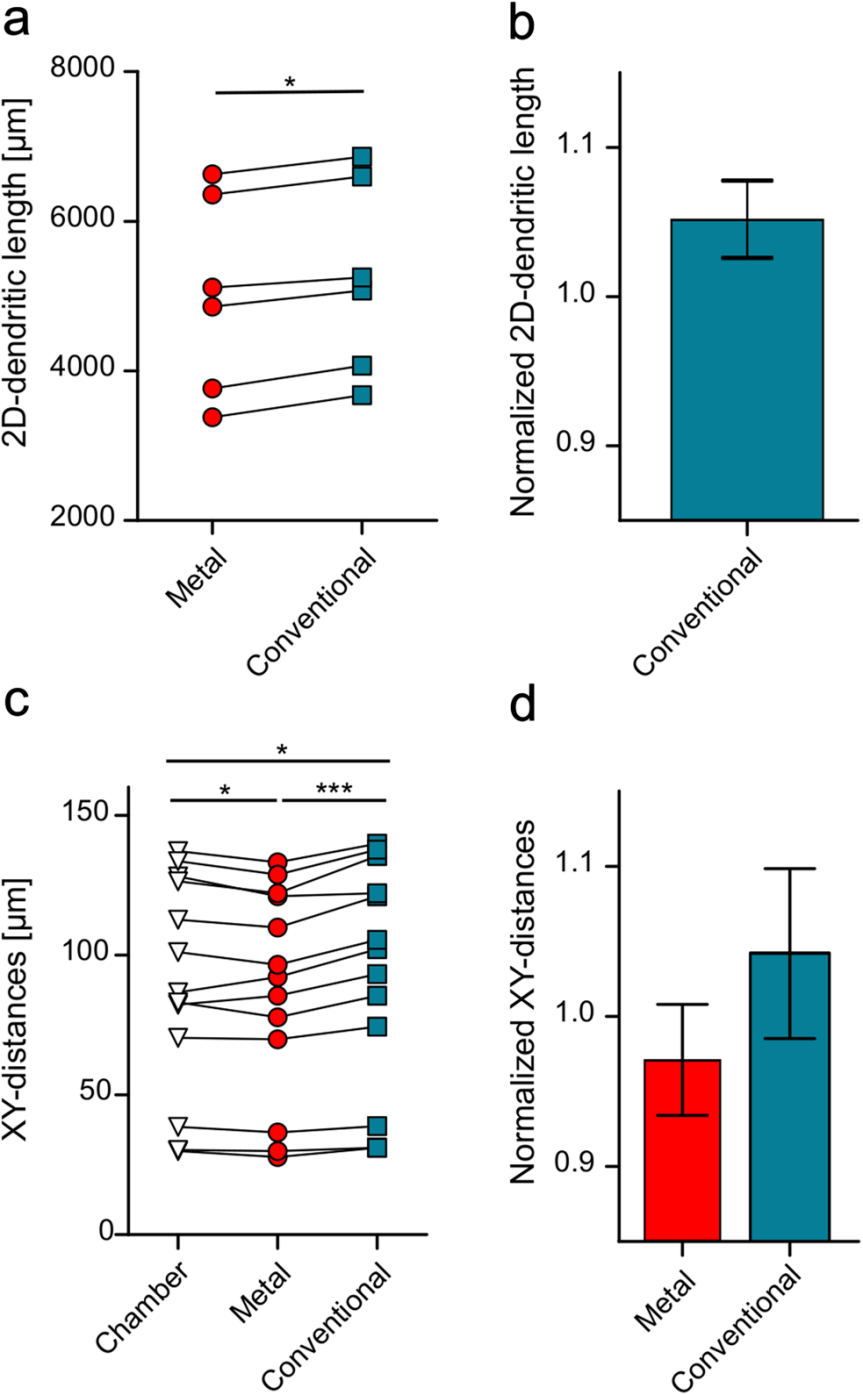
Dimensional changes in the *xy*-plane of the slices after embedding. **a** Summary plot of the 2D-dendritic lengths of 6 neurons measured in the *xy*-dimensions of reconstructions made with metal spacer (red circles) and after re-embedding without spacer (blue squares). **b** Normalized dendritic length in the *xy*-dimensions of reconstructions made after conventional embedding (blue bar) calculated relative to the length in the reconstructions with metal spacer. **c** Summary plot of distances between landmarks (13 pairs in 4 slices) in the *xy*-plane measured in photomicrographs of the slices made in the recording chamber (open triangles), at day 4 after embedding with the metal spacer system (red circles) and 14 days after re-embedding without spacer (blue squares). **d** Summary bar chart of landmark distances in the *xy*-plane measured in the slices made with the metal spacer (red bar) and after re-embedding without spacer (blue bar) normalized to the corresponding distances in the recording chamber (16 sets of distances in 4 slices). Error bars indicate standard deviation; statistical significance: *p < 0.05, ***p < 0.001

Next, we tested if the reconstructions showed evidence of deformation in the *xy*-plane. For this, we first projected all reconstructions into the *xy*-plane by setting all *z* values to zero, thus, reducing them from 3D to 2D ones. In these flattened reconstructions, we repeated the comparison of the 2D-dendritic lengths between the two consecutive reconstructions for each neuron. The comparison revealed that in the second reconstructions, made after the re-embedding with no spacer, the total 2D-dendritic lengths was consistently larger by a factor of 5.2 ± 2.3% on average than in the first ones (*p* = 0.03, Wilcoxon signed rank test, six neurons; Fig. 4a, b), suggesting that while the slices suffered a substantial shrinkage along the *z*-axis, they also showed a moderate dilation in the *xy*-plane after re-embedding with the conventional method.

To confirm this result, we made measurements of dendritic segments in the pairs of confocal image stacks and compared their *xy*-dimensions for the two embedding states: in the second set of image stacks with no spacer, the sampled dendritic segments had larger *xy*-dimensions when compared to their dimensions in the first set of image stacks made with the metal spacer by a factor of 8.2 ± 6.1% on average (*p* < 0.0001, Wilcoxon signed rank test, 110 dendritic segments from 4 pairs of image stacks, Online Resource 3a, b).

Finally, to assess the dilation of the slices directly, we have made photomicrographs of a set of slices, first, in the recording chamber, second, at day 4 after embedding with the metal spacer and, finally, 14 days after re-embedding the slices without a spacer. Measurements of distances between landmarks in the plane of the slices (i.e. in the *xy*-plane) showed that their dimensions were minimally reduced, by a factor of 2.9 ± 3.6% on average, when they were mounted and imaged with the metal spacer relative to their dimensions in the recording chamber (*p* = 0.01, Wilcoxon signed rank test, 16 sets of measurements in 4 slices; Fig. 4c, d). In contrast, when re-embedded without a spacer, the slices showed a dilation of 4.2 ± 5.5% relative to their dimensions in the recording chamber (*p* = 0.02, Wilcoxon signed rank test, Fig. 4c, d).

In summary, our analysis of the neuronal morphologies obtained with the two different embedding method demonstrate that shrinkage with conventional embedding is not uniform along the *z*-axis and the slices suffer additional deformation in the form of dilation in the *xy*-plane. Therefore, the use of a linear correction factor applied to the *z*-axis cannot properly compensate for morphological errors produced by the shrinkage process and may even accentuate some aspects of the deformations observed with conventional embedding.

## Discussion

Here, we describe a new method for the embedding of acute brain slices with intracellularly labeled neurons and show that the use of a metal spacer can effectively minimize shrinkage along the *z*-axis. We further demonstrated that shrinkage with conventional embedding develops rapidly and leads to a substantial reduction in the thickness of the slices. Moreover, this massive shrinkage along the *z*-axis is non-linear and accompanied by dilation in the *xy*-plane. The impact of these non-uniform deformations on the morphology and anatomical parameters cannot be compensated by linear correction.

### Rapid and strong shrinkage of slices with conventional embedding

Histological procedures, which include drying and dehydration, have been shown to produce severe shrinkage of tissue samples (Pyapali et al. 1998; Hellwig, 2000; Marx et al. 2012) unless the samples are postfixed using for example osmium tetroxide to stabilize lipid membranes. A disadvantage of postfixation with osmium, however, is that it leads to strong opacity of the tissue, precluding light microscopic investigations of thicker samples, such as the 300–400 µm acute brain slices used in combined electrophysiological and morphological investigations. While re-sectioning of the slices offers a solution to this problem, the time and workload involved is prohibitive for large series of samples and not justified, unless light microscopy is routinely coupled with subsequent electron microscopic analysis (Gulyas et al. 1993; Vida et al. 1998).

Elimination of dehydration steps, combined with mounting in an aqueous media, can reduce shrinkage during histological processing. Protocols based on this approach for the visualization of neurons in acute slices have been widely established as a more efficient alternative. However, this approach cannot prevent shrinkage of the slices after embedding (Egger et al. 2008; Swietek et al. 2016). Indeed, our data show that conventional embedding of slices, as whole mounts without spacer, in aqueous media results in severe shrinkage along the *z*-axis. The slices suffer a substantial initial shrinkage already within a few hours after coverslipping. The z-shrinkage continues at a high rate in the first days after embedding, but progressively slows down in the following weeks and reaches an asymptotic value of approximately 50% beyond 2 months. Reasons for this shrinkage are most likely the weight of the coverslip and adhesive forces between the glass surfaces and the mounting medium. Additionally, loss of water content can also contribute in the long term. The effect of this deformation on cellular morphology is a function of the direction of neurites: ones which run perpendicular to the slice surface suffer the strongest reduction in length (Egger et al. 2008). Thus, depending on the orientation of the dendrites and axons, some cells will be more affected by this artifact than others.

To prevent shrinkage, an agar spacer has been introduced between slide and coverslip in previous studies (see e.g. Degro et al. 2015). However, we find now that, using such a spacer, one can avoid the initial shrinkage, but cannot prevent it in the long term. In fact, the shrinkage of slices with agar spacer beyond 3–4 days becomes even stronger than in slices embedded without spacer. The dramatic long-term shrinkage (65–75%) with agar spacer was an unexpected finding of our study and the reason for it is not clear. A possible explanation is that water loss of the agar over time could lead to volume loss of the spacer, producing additional adhesive forces affecting the slice. Therefore, we recommended the use of agar spacer only if imaging is performed within a couple of hours after embedding, as a simple and efficient way of temporarily preventing slice deformation. If used in this manner, agar spacer is a cost effective alternative to the metal spacer, as the latter incurs material and production costs.

### Non-uniform deformation of embedded slices affects neuronal morphology and anatomical parameters

Assuming a uniform shrinking process, attempt has been made to compensate arising morphological deformation by applying a linear correction factor to 3D reconstructions (Pyapali et al. 1998; Hellwig 2000; Marx et al. 2012; Degro et al. 2015). However, it is unknown if this compensation is adequate at all. By imaging and reconstructing the same cells twice, first with a metal spacer followed by an embedding without a spacer, we examined the impact of z-shrinkage on neuronal morphology and found evidence for a non-uniform shrinkage along the *z*-axis and an associated dilation in the *xy*-plane. These distortions significantly compromise the fidelity of 3D-reconstructions and the derived morphological parameters in a way that cannot be corrected by linear methods: first, our results show that the strongest shrinkage along the *z*-axis occurs closest to the top and bottom cut surfaces of the slices. Non-linear, non-uniform shrinkage in thin brain sections has been previously described in stereological studies (e.g. Gardella et al. 2003). More relevantly, Egger et al. (2008) observed a similar pattern of non-linear shrinkage in acute cortical slices, by comparing neuronal structures imaged first in vitro and subsequently after a conventional embedding procedure using aqueous mounting medium. A major reason for the non-uniform shrinkage can be that the superficial parts of the acute slices are more compressible than deeper tissue, due to the damage caused by the slicing procedure and the subsequent washout of debris from these damaged layers (Egger et al. 2008). Additionally, adhesive forces may differentially affect superficial and deep parts of the slices once they are mounted. Thus, while linear models, as a first-order approximation, may help to assess shrinkage-induced morphological errors (Egger et al. 2008), they cannot fully eliminate these errors and will also exaggerate differential deformation by undercompensating *z*-axis shrinkage in superficial and overcompensating it in the deep regions.

Second, our findings reveal that a moderate degree of dilation in the *xy*-plane additionally impacts morphological parameters, such as the dendritic length resulting in an opposing effect. Beside similar histological procedures and embedding, Egger et al (2008) did not find such dilation. This could be due to the different mounting medium used (Mowiol) in their study. This is supported by findings that, depending on the precise composition of clearing solutions used, tissue samples may show volume expansion, shrinkage, or maintain their volume (Hama et al. 2011, 2015; Kuwajima et al. 2013; Richardson and Lichtman, 2017). The dilation, on one hand, can partially mask the effect of shrinkage. On the other hand, it results in a systemic overestimate of dendritic length, when the linear correction is applied to the *z*-axis. Measurements of the dimensions of the slice before and after embedding can help to estimate the degree of dilation and enabling partial corrections. However, as with *z*-axis shrinkage, these may also suffer from confounding effects of possible non-linearities. In summary, our findings of combined and non-uniform deformations of the slices argue for the need of preventing shrinkage rather than attempting to compensate it.

### Metal spacer can efficiently and stably minimize shrinkage of embedded brain slices

Embedding procedure with metal spacers can not only prevent the initial shrinkage directly after embedding, but also strongly minimizes the shrinkage process in the first days post-embedding. Thus, if cells were imaged in that time window, one can achieve minimally-compromised neuronal morphologies and reconstructions. Shrinkage correction is not necessary, as there is only negligible morphological distortion below 5% along *z*-axis and less than 3% in the *xy*-plane. However, the metal spacer system cannot fully prevent slices from shrinkage in the long term, reaching a moderate but significant value of 16.3 ± 8.9% after two months. As the compression by the coverslips as shrinkage contributor is eliminated, ongoing shrinkage could be cause by the embedding medium. It was previously discussed, that many embedding media, including most aqueous mounting media, enable z-shrinkage and only 100% glycerol could fully prevent shrinkage (Claiborne et al. 1986; Mainen et al. 1996). However, when using pure glycerol, it is difficult to seal and handle the slides properly, as medium remains fluid. The slice in its mounting medium can move while imaging with a confocal microscope, leading to compromised images. Long-term storage of mounted slides is a further issue, as the mounting medium evaporates slowly over time. Finally, stained neurons tend to fade in glycerol over time; this effect being noticeable already within weeks (Jaeger 2000). Considering these disadvantages 100% glycerol is not a convenient alternative for embedding.

In summary, slices suffer a marked, non-linear shrinkage along the *z*-axis and associated dilation in *xy*-plane after conventional embedding procedures, causing distortion of neuronal morphology and producing errors in derived anatomical parameters. As these deformations are non-compensable with simple, linear approaches, minimizing these effects is imperative. The metal spacer system can efficiently and stably minimize these artifacts, particularly within the first days post-embedding, and, thereby, provides a substantial improvement for morphological investigations of intracellularly labeled neurons in acute slices.

## Electronic supplementary material

Below is the link to the electronic supplementary material.

Online Resource 1 (Supplement to Fig. 1) The impact of shrinkage on estimates of dendritic spine density. **a** Summary plot of spine densities calculated from image stacks projected onto the *xy*-plane from slices embedded with metal spacer (red bar) and after re-embedding without spacer (cyan bar). **b** *XY*-projection of a representative dendritic segment embedded first with the metal spacer (top) and after re-embedding following the conventional approach without a spacer (bottom). **c** Corresponding summary plot of spine densities calculated from image stacks projected onto the *yz*-plane for the two embedding states. **d** *YZ*-projection of the same dendritic segment as in panel **b** embedded with the metal spacer (top) and after re-embedding with the conventional approach (bottom). The latter image was compensated for shrinkage for better comparison. **e** Summary plot of spine densities calculated 3-dimensionally in the image stacks obtained with metal spacer (red bar) and after re-embedding without spacer (cyan bar). Error bars indicate standard deviation; statistical significance: * *p*< 0.05. (TIF 450 kb)

Online Resource 2 (Supplement to Fig. 3) The impact of shrinkage on anatomical parameters derived from reconstruction of intracellularly labeled neurons using the Neutube software package. **a** Plot of the total dendritic lengths of 6 neurons measured in reconstructions made with metal spacer (red circles), in reconstructions made after re-embedding without spacer (blue squares) and after linear shrinkage correction was applied to the second set of reconstructions (cyan triangles). Lines connect data points representing measurements from the same neuron. **b** Summary bar chart of the dendritic lengths measured in the reconstructions made after conventional embedding (blue bar) and after linear shrinkage correction was applied (cyan bar), normalized to the corresponding lengths measured in reconstructions with metal spacer. Error bars indicate standard deviation; statistical significance: * *p*< 0.05. (TIF 210 kb)

Online Resource 3 (Supplement to Fig. 4) Deformation of brain slices in the *xy*-plane with conventional embedding. **a** Plot of *xy*-dimensions of dendritic segments (110 dendritic segments from 4 slices) measured in confocal images obtained in slices embedded first with a metal spacer (red circles) and after re-embedding following the conventional approach (blue squares). Lines connect data points corresponding to the same dendritic segment. **b** Length of dendritic segments in the xy-plane of the image stacks obtained after re-embedding without spacer (blue bar) were normalized to the values obtained with the metal spacer. Error bar indicate standard deviation; statistical significance: *** *p*<0.0001. (PDF 76 kb)
