# Supplementary figures and images for: Minimizing shrinkage of acute brain slices using metal spacers during histological embedding
Source: Brain Struct Funct. 2020 Sep 12;225(8):2577–89. doi: 10.1007/s00429-020-02141-3 (PMC7544706; doi:10.1007/s00429-020-02141-3)

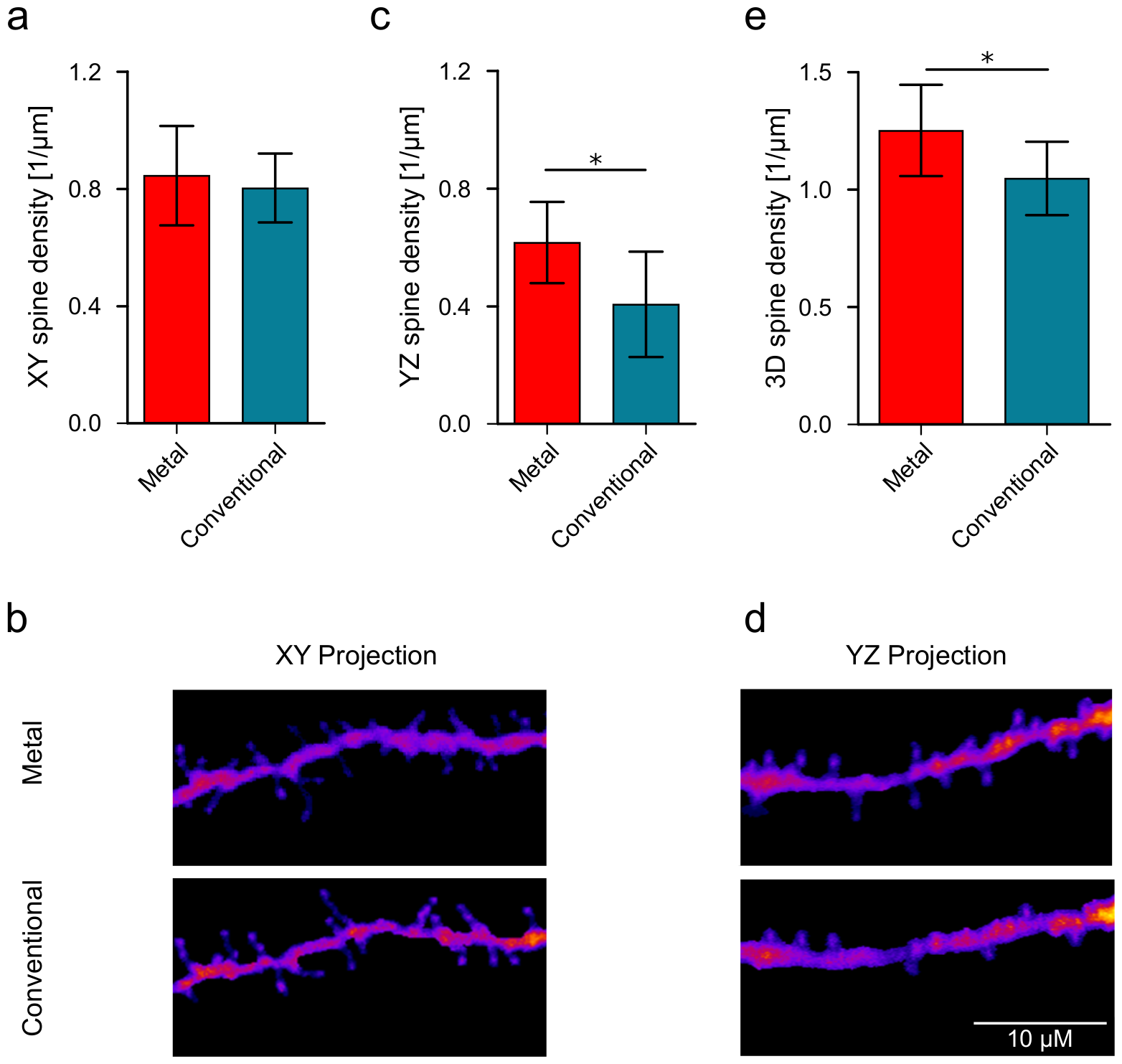

Supplement: Supplementary file 1 — Online Resource 1 (Supplement to Fig. 1) The impact of shrinkage on estimates of dendritic spine density. a Summary plot of spine densities calculated from image stacks projected onto the xy-plane from slices embedded with metal spacer (red bar) and after re-embedding without spacer (cyan bar). b XY-projection of a representative dendritic segment embedded first with the metal spacer (top) and after re-embedding following the conventional approach without a spacer (bottom). c Corresponding summary plot of spine densities calculated from image stacks projected onto the yz-plane for the two embedding states. d YZ-projection of the same dendritic segment as in panel b embedded with the metal spacer (top) and after re-embedding with the conventional approach (bottom). The latter image was compensated for shrinkage for better comparison. e Summary plot of spine densities calculated 3-dimensionally in the image stacks obtained with metal spacer (red bar) and after re-embedding without spacer (cyan bar). Error bars indicate standard deviation; statistical significance: * p< 0.05. (TIF 450 kb) [file 429_2020_2141_MOESM1_ESM.tif]

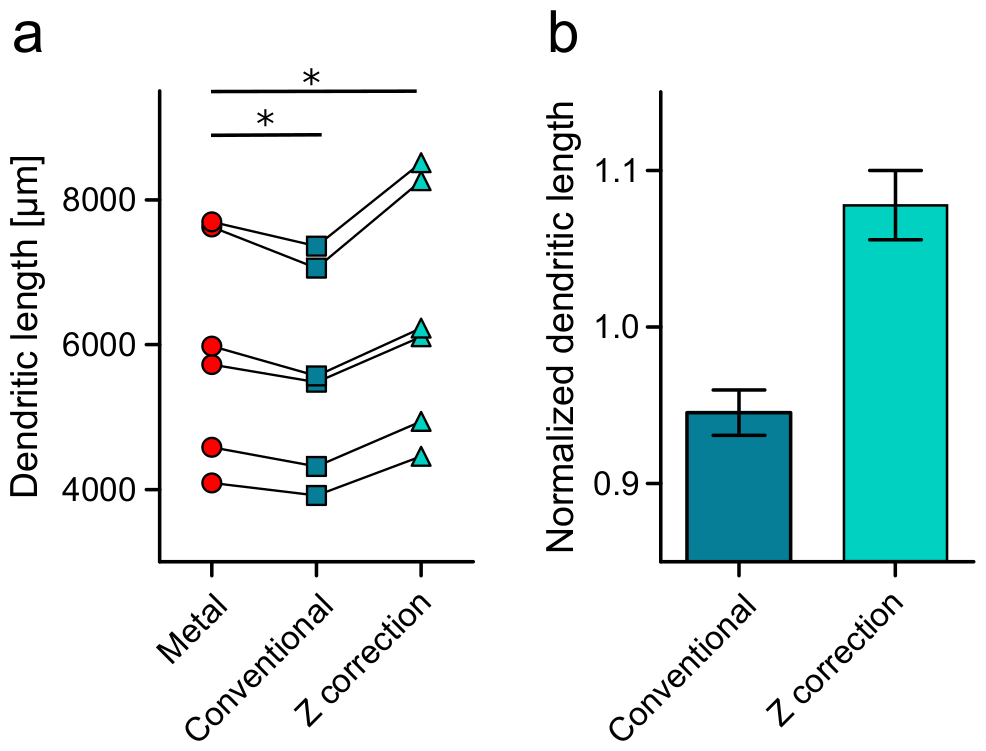

Supplement: Supplementary file 2 — Online Resource 2 (Supplement to Fig. 3) The impact of shrinkage on anatomical parameters derived from reconstruction of intracellularly labeled neurons using the Neutube software package. a Plot of the total dendritic lengths of 6 neurons measured in reconstructions made with metal spacer (red circles), in reconstructions made after re-embedding without spacer (blue squares) and after linear shrinkage correction was applied to the second set of reconstructions (cyan triangles). Lines connect data points representing measurements from the same neuron. b Summary bar chart of the dendritic lengths measured in the reconstructions made after conventional embedding (blue bar) and after linear shrinkage correction was applied (cyan bar), normalized to the corresponding lengths measured in reconstructions with metal spacer. Error bars indicate standard deviation; statistical significance: * p< 0.05. (TIF 210 kb) [file 429_2020_2141_MOESM2_ESM.tif]

## Suppl. 3

**a**

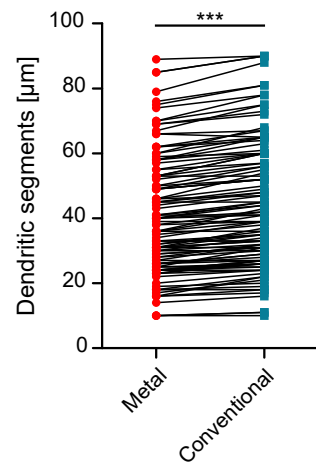

**b**

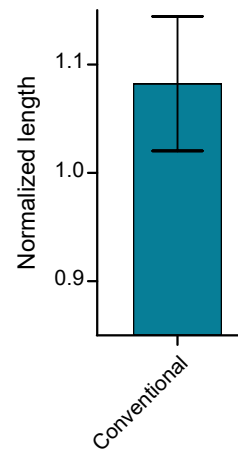

Supplement: Supplementary file 3 — Online Resource 3 (Supplement to Fig. 4) Deformation of brain slices in the xy-plane with conventional embedding. a Plot of xy-dimensions of dendritic segments (110 dendritic segments from 4 slices) measured in confocal images obtained in slices embedded first with a metal spacer (red circles) and after re-embedding following the conventional approach (blue squares). Lines connect data points corresponding to the same dendritic segment. b Length of dendritic segments in the xy-plane of the image stacks obtained after re-embedding without spacer (blue bar) were normalized to the values obtained with the metal spacer. Error bar indicate standard deviation; statistical significance: *** p<0.0001. (PDF 76 kb) [file 429_2020_2141_MOESM3_ESM.pdf]
